# Enhancing Cognitive Ergonomics in Robotic Surgery—A Narrative Review

**DOI:** 10.3390/s26216726

**Published:** 2026-10-23

**Authors:** Avni Agarwal, Zhen Hao Ang, Shing Wai Wong

**Affiliations:** 1 Randwick Campus, School of Clinical Medicine, The University of New South Wales, Sydney, NSW 2031, Australia; 2 Department of General Surgery, Prince of Wales Hospital, Barker Street, Randwick, Sydney, NSW 2031, Australia

**Keywords:** robotic surgery, ergonomics, cognition

## Abstract

Robotic-assisted surgery (RAS) has transformed minimally invasive procedures by improving physical ergonomics, surgical field visualisation and instrument manipulation. However, RAS introduces unique cognitive load for the surgeon. Cognitive ergonomics aims to enhance the mental resources to optimise the interactions between surgeons and their environment, thereby improving the wellbeing and performance of surgeons and patient outcomes. Current evidence highlights that RAS redistributes, rather than eliminates, ergonomic burden from the physical to the mental domain. Subjective measurement tools remain the most widely used; however, physiological measures offer continuous and real-time data regarding cognitive workload fluctuations during surgery. Strategies to improve cognitive ergonomics include surgeon-level strategies, interface and system design alterations, operating room workflow optimisation, incorporation of artificial intelligence, and training and education. This narrative review summarises the current evidence regarding the mental demands associated with RAS, cognitive workload measures, the consequences of poor cognition, and emerging strategies to improve cognitive ergonomics in RAS.

## 1. Introduction

The introduction of robotic platforms such as the da Vinci^®^ Surgical System has revolutionised minimally invasive surgery. Robotic-assisted surgery (RAS) provides several advantages, including high-definition three-dimensional (3D) visualisation of the operative field and improved instrument manipulation [1]. Yet, despite the widespread adoption of RAS, evidence regarding its impact on the surgeon remains limited. A paradox exists within the current literature: whilst RAS may reduce the physical demands associated with the laparoscopic approach, it leads to a shift towards increased cognitive demands. This increased cognitive load can significantly impact the surgeon’s decision-making, performance, and consequently, patient outcomes.

Cognitive ergonomics aims to optimise surgical performance by evaluating the mental resources required to perform tasks [2]. Cognitive workload refers to the mental effort required to complete a task [3]. This differs from stress or physical workload. Stress represents the psychological and physiological response to a demanding situation where physical workload represents the biomechanical demands to perform a task [2]. Working memory temporarily handles information in any given moment, but has limited capacity and can only retain that information for approximately 20 s, unless it is further consolidated into long-term memory by active rehearsal or repetition [4]. On the other hand, long-term memory has the capacity to store unlimited information over extended periods [4]. The theoretical framework establishing this load can be defined through the cognitive load theory (CLT), which divides workload into three domains: intrinsic load, extraneous load, and germane load [5]. Intrinsic load is the load that is determined by the complexity of the task, extraneous load is the load influenced by the external environment, and germane load is the load dedicated to improving performance by using appropriate resources [5]. During RAS, intrinsic load is increased due to the sustained complexity of the console and surgical task, whilst extraneous load may also increase due to the loss of haptic feedback and altered team dynamics [6]. Cognitive overload occurs when a task exceeds the capacity of working memory, impairing surgical performance [7]. With greater experience and practice, surgeons develop automaticity, allowing them to perform tasks with less conscious effort [7].

Approximately 25% of the variation in patient outcomes can be attributed to surgical performance and skill [8]. When optimal cognitive workload is balanced with maximal technical skills, surgical performance is associated with fewer errors and improved patient outcomes [7]. The Yerkes–Dodson curve suggests the downstream effects of both cognitive under-stimulation and overload, and RAS amplifies cognitive load through its unique technical and environmental demands [9]. Task performance is optimised when surgeons are experiencing a moderate level of mental arousal, with simple tasks benefitting from slightly higher levels of alertness, and complex tasks performed more effectively under lower levels of arousal [1].

The importance of understanding cognition in RAS has increased, as emerging technologies reshape the surgical experience. Artificial intelligence (AI) is being integrated into robotic surgery to support decision-making, risk prediction, anatomical recognition, and enhance visual information [10]. Telesurgery offers novel applications in remote care; however, it remains limited by transmission latency and altered situational awareness [11]. Despite the potential of RAS to improve surgical precision, efficiency, and overall patient outcomes, it may affect cognitive workload by changing how surgeons process information, make decisions, and interact with their environment.

This narrative review aims to summarise the most contemporary evidence on the impact of robotic surgery on the cognitive ergonomics of the operating surgeon. It will also summarise the various measurements of cognition as well as strategies to reduce cognitive load in robotic surgery.

## 2. Methods

A literature search was conducted using PubMed, Embase and Scopus from inception to June 2026. The following keywords and MeSH terms were combined using Boolean operators: (“robotic surgery” OR “robot-assisted surgery”) AND (“cognitive ergonomics” OR “cognition” OR “cognitive measure” OR “cognitive fatigue”). A separate database search was conducted for measurement tools of cognitive load. Titles and abstracts were screened by two of the authors (AA and ZHA). Only studies published in English language were included. Reference lists of the identified articles and key review papers were also manually screened for additional studies that were missed from the initial search. As this is a narrative review, the protocol was not registered with PROSPERO and a PRISMA diagram was not included. No formal risk-of-bias assessment was performed.

## 3. Ergonomic Considerations Across Different Surgery Types

### 3.1. Open Surgery

Open surgery provides less restricted instrument movement and direct visualisation of anatomical structures, providing surgeons with complete haptic and proprioceptive access within the operative field [12]. However, it is associated with significant physical demands on the surgeon [12]. Open surgery is associated with high cognitive demands, reflected by increased autonomic nervous system activation [13]. Cognitive workload is influenced by an interaction between procedural complexity, physiological stress, and physical exertion, rather than the surgical approach alone [13].

### 3.2. Laparoscopic Surgery

Laparoscopic surgery introduces unique ergonomic constraints that influence the interactions between the surgeon, instruments, and patient. Laparoscopic instruments are non-wristed, limiting movement to four degrees of freedom within a single port [14]. In addition, the vast majority of conventional laparoscopic systems utilise two-dimensional monitors, reducing depth perception and causing the surgeon to rely on compensatory visual mechanisms [15]. Furthermore, the fulcrum effect causes the hands to move in the opposite direction to the instrument tips, increasing the complexity of instrument manipulation [16]. Alongside these technical constraints, surgeons are also required to maintain non-ergonomic postures for prolonged durations which contribute to increased musculoskeletal strain, especially in the back, neck, shoulders, and lower extremities [6].

Increased cognitive workload is associated with demands on spatial awareness, hand–eye coordination, reduced movement range, and spatial limitations. Laparoscopic procedures introduce visual–motor dissociation, as surgeons view the operative field through a two-dimensional image on a monitor, and operate using their hands [17]. This causes misalignment between the visual axis and the forearm–instrument motor axis, thereby introducing ergonomic limitations [17]. A study found that laparoscopic tasks induced greater brain activity in the prefrontal cortex, reflecting the increased mental effort [18].

### 3.3. Robotic Surgery

RAS reflects an evolution in minimally invasive surgery, addressing many of the ergonomic limitations associated with both open and laparoscopic procedures. The widely used da Vinci^®^ Surgical System employs a closed-console system design, in which the surgeon operates with their head enclosed in a console while communicating with the theatre team via an integrated microphone [19]. The da Vinci^®^ Surgical System improves the physical ergonomic profile of minimally invasive surgery by providing immersive 3D binocular vision and allowing surgeons to remain seated while operating [6]. Its seven degrees of wrist movement closely replicate the human hand motion range, and the integrated tremor filtration and motion scaling improve precision [20,21,22]. Whilst RAS reduces upper extremity strain, physical burden shifts to the neck, trunk, and forearms, reflecting redistribution rather than complete elimination of ergonomic demands [6].

RAS has been suggested to reduce working memory demands, allowing surgeons to focus on higher-order functions such as decision-making and procedural planning [23]. However, it introduces other cognitive challenges that differ from those experienced in laparoscopic surgeries. For instance, cognitive demand in RAS is more sustained throughout the procedure rather than being transient, thereby shifting ergonomic challenges from the physical to the mental domain [6].

The absence of haptic feedback and tactile sensation in RAS increases reliance on visual information, resulting in a phenomenon referred to as “visual haptics” [24]. Tactile feedback is crucial for tissue retraction, dissection, and manipulation, but earlier generations of the da Vinci^®^ Surgical Systems lack haptic feedback [25]. Thus, surgeons are required to use visual cues to assume tactile properties of tissues, increasing mental demands and cognitive fatigue [25]. With experience, surgeons can learn to use visual cues such as tissue compression to compensate for the loss of haptic feedback [25].

RAS requires surgeons to simultaneously control multiple instruments, with three instrument arms, therefore dividing attention and increasing mental multitasking [26]. Each instrument also has different grip strengths and lengths of articulating tips, requiring surgeons to continuously adapt their motor control strategies [26]. Additionally, the surgeon’s forearms are placed on the console bar, while simultaneously using foot pedals to switch control between different robotic arms, introducing another layer of coordination that increases mental demands [26,27]. Furthermore, unlike laparoscopic procedures, robotic surgeons frequently control the endoscopic camera themselves, creating an additional mental task that increases cognitive workload [26]. However, the steady view reduces cognitive demands [28]. Finally, the master controllers only move within a limited physical range, and when the surgeon reaches the end of their comfortable range of motion, they press a clutch to reposition their hands [29]. Although these interruptions are brief, the accumulation of these task disruptions and repositioning processes adds to the cognitive demands [26].

Cognitive workload generally increases with both the duration and complexity of the surgery. The majority of simulation studies report lower subjective mental workload during robotic procedures, which may be related to the fact that the tasks are shorter and less complex, reducing physical strain [23]. In clinical settings, surgeries are longer and more complex. This leads to increased muscle workload where more motor neurons are activated and the firing rate increases to maintain muscle contraction, resulting in a greater perceived effort [6,30,31]. RAS usually takes longer to complete, allowing fatigue to accumulate over this time [32]. One study found that prolonged robotic activity significantly worsened surgical performance, with deterioration particularly evident after approximately two hours of console activity [32]. Another study found that the primary surgeon experienced the greatest fatigue accumulation during RAS, attributed to a prolonged static muscle activation, and sustained concentration and decision-making burdens [33]. A systematic review further found that fatigue affects cognitive processes more than technical skills because routine movements become automatic after years of practice, but complex decision-making remains case-specific [34]. Consequently, although robotic systems reduce physical demands, cognitive demands paradoxically increase, causing mental strain, stress, and burnout [34].

Closed-console systems reduce situational awareness, as the surgeon is not at the bedside [19]. Microphones in the closed-console systems pick up background conversations easily, requiring staff to specifically discuss case-relevant communication, further reducing distractions [19]. In addition, non-verbal communication such as gestures, eye contact, and pointing is removed, causing staff to be more explicit and clearer with instructions; however, this reduces accuracy and efficiency of communication [19]. Therefore, open-console robotic surgery has been associated with improved team dynamics when compared with closed-console systems; however, this is dependent on team experience [19].

## 4. Cognitive Workload Measures

Understanding cognitive workload during surgery is crucial to improving performance and outcomes. Measurement remains multifactorial and complex. Performance is typically assessed through either measuring physical or cognitive workload; however, this framework can oversimplify the interdependent nature of these demands [35]. Workload measurement can be classified through subjective self-reporting tools, physiological measures, and performance-based modalities. An ideal measurement approach should be continuous, non-invasive, sensitive, ecologically valid, and specific. Currently no single modality satisfies all criteria simultaneously [36,37]. Table 1 compares various cognitive workload measurement techniques in surgery.

Subjective tools remain the most widely implemented technique to measure surgical workload. Self-reporting tools were commonly used in studies, with the NASA-TLX being the most common [37]. The NASA-TLX is a multidimensional measurement tool that analyses six workload demands including, mental, physical, temporal, performance, effort, and frustration, to yield a score from 0 to 100 that reflects perceived workload during a procedure [37]. A prospective observational study demonstrated that the NASA-TLX scores correlated with the surgeons’ subjective experience of the surgery, and was able to differentiate workload between different surgeries performed by the same surgeon [38]. However, the NASA-TLX lacks surgery-specific sensitivity, which can be addressed using the SURG-TLX, an adaptation of the NASA-TLX [39]. The SURG-TLX analyses surgery-specific demands rather than only cognitive load, including mental demand, physical demand, temporal demand, task complexity, distractions, stress, and situational demands, providing greater relevance to surgical contexts [39]. This expanded framework allows for identification of which workload aspect is increased rather than reducing workload into a single global score [39].

Subjective tools such as the NASA-TLX and SURG-TLX are beneficial given their wide utility and research across both surgical and industrial ergonomics. Their questionnaire-based approach makes them accessible, feasible, and prevents interference with the surgical workflow [39]. However, the self-reporting technique relies on retrospective self-reflection, leading to recall and emotional bias [39,40]. Additionally, surgical demands vary throughout a procedure, preventing retrospective approaches from capturing real-time fluctuations in cognitive load and fatigue [39]. Comparative studies further demonstrate discrepancies between physiological and subjective measurements, with a study reporting no difference in cognitive fatigue between laparoscopic and robotic surgery when using the NASA-TLX, but physiological heart rate metrics identifying lower fatigue during RAS [41].

Advances in wearable technology have enabled continuous physiological monitoring without interrupting surgical workflow [42]. Heart rate variability (HRV) is a physiological indicator of the autonomic nervous system (ANS) activity, which responds to stress [40,43]. Sympathetic activation has chronotropic and inotropic effects on the heart, causing vasoconstriction, increased blood pressure, increased respiratory rate, and shallow breaths, while parasympathetic activation resulting in the opposite effects [13]. HRV as a reflection of the autonomic nervous system activation represents the surgeon’s comfort, dexterity, and stress during procedures [13]. Sympathetic activation increases when experiencing stress, reducing time and variability between heartbeats. Therefore, a lower HRV indicates greater levels of physiological stress [40,43]. This elevated sympathetic activation further suppresses HRV metrics, such as standard deviation of normal-to-normal intervals (SDNN) and root mean square of successive differences (RMSSD) [44]. Increased stress levels are associated with reduced surgical performance and higher rates of potential adverse events, but there is a bidirectional relationship with adverse events inducing stress and errors [43,45]. A study found that there was a strong correlation between surgeons’ subjective experiences of stress and HRV, with more technically difficult tasks inducing greater cognitive load [46].

HRV can be measured using wearable devices, preventing workflow disruption intraoperatively, making it a useful objective marker of cognitive workload [40]. A study found that chest and wrist devices that measure heart activity are generally comfortable and do not cause significant distraction [47]. Electrocardiogram (ECG) devices such as the Polar H10 chest strap produced the most reliable data, and is feasible and available [47]. However, HRV is insensitive to primary task differences and cannot discriminate between sources of cognitive load, which are crucial to ergonomic and interventional designs [48]. Additionally, HRV is confounded by differences in gender, physical factors such as exercise, and social factors such as caffeine and alcohol [46]. Multitasking such as talking, which influences respiratory patterns, may also influence HRV measurements [48].

Neurophysiological measures such as electroencephalograph (EEG) achieves 100% sensitivity when detecting cognitive demand and can instantaneously detect changes in brain activity, differentiating between different sources of load, including intrinsic, extraneous, and germane [36,48]. Additionally, increased frontal midline theta power is associated with increases in cognitive workload and working memory, and suppression in alpha waves reflect increased attentional processing during demanding tasks [49]. However, a study found that more complex EEG devices worn on the scalp and forehead are often uncomfortable, distracting, can induce headaches, and connections can be disrupted, producing unreliable data [47].

Functional near-infrared spectroscopy (fNIRS) represents another neurophysiological measurement modality. fNIRS measures oxygen levels in the blood by shining near-infrared light into the brain, measuring brain activity through a non-invasive and wearable device [50]. Areas of the brain, particularly the prefrontal cortex, that are involved in greater cognitive functioning will require more oxygen, therefore increasing blood flow to that area [51]. fNIRS has lower sensitivity to body movements, is portable, safe, and wearable, making it superior to traditional EEG approaches [52]. However, it has lower temporal resolution, only measuring activity in the cortex, may be confounded by physiological changes such as blood flow alterations unrelated to brain activity, and motion may still introduce artefacts [52].

Eye tracking is a real-time measure of workload using wearable eye-tracking glasses or microscopic oculars; however, its interpretation remains complex [53]. Higher cognitive workload and increased task complexity is associated with increased pupil dilation diameter and blink rate, or task-evoked pupillary response [54]. Critical flicker fusion frequency (CFFF) is the frequency at which flickering light is first perceived as continuous [55]. CFFF indirectly measures cognitive function as it measures temporal visual processing [55]. However, despite its objective and simple utility, CFFF can be influenced by biological and environmental factors, and should be interpreted alongside other measurement tools [55,56]. A systematic review reported that increased blink rate was associated with increased cognitive workload [57].

Salivary alpha-amylase and cortisol are two commonly used biomarkers for physiological stress and are indicators of sympathoadrenal medullary and hypothalamic–pituitary–adrenal axes [58]. A study compared saliva samples in both robotic and laparoscopic procedures, with RAS producing a lower increase in salivary amylase, but no statistical significance when comparing salivary cortisol [58]. However, increases in amylase versus cortisol may reflect different psychosocial processes and are influenced by circadian rhythms, caffeine, diet, infection, and the time of day, limiting application as a physiological stress marker [58,59,60].

Performance-based measures, such as operative duration, intraoperative blood loss, postoperative complications, and hospital length of stay, have traditionally been used as methods to evaluate surgical outcome and complexity [38]. Surgical outcomes can be measured by both surgical complications during the operation, or by future consequences, and the number of errors can be reduced by evaluating surgical performance [7]. Performance-based metrics provide objective and standardised clinical outcomes that can be measured [38]. However, they fail to capture the surgeon’s subjective and subconscious experience of the procedure, and variations of many outcomes can be attributed to patient factors [38]. Performance-based measures may also be influenced by equipment malfunctions, workflow inefficiencies, or surgical experience [38]. Studies have demonstrated that the NASA-TLX scores do not consistently correlate with performance-based metrics, as these factors are influenced by patient-dependent variables [38]. Additionally, automated performance metrics generated by robotic surgical systems may assess technical performance, but again, fail to capture the cognitive demands experienced by surgeons [61].

A systematic review further highlights that patient outcomes remain a poor measure of cognitive workload because complications can occur due to patient-specific health, and peri- and postoperative management [34]. When surgeons recognise that they are mentally fatigued, they invest more cognitive effort to stabilise their performance, thereby increasing their cognitive workload [34,62]. Therefore, patient outcomes are an indirect measure of cognitive workload as surgeons compensate for fatigue [34]. Additionally, Fitts’ Law describes a trade-off relationship, where faster movements can occur at the expense of accuracy, whilst slower movements may improve precision [63]. However, experienced surgeons who have developed automaticity can perform the same technical movements with greater speed and accuracy, even during periods of high cognitive workload [2]. Consequently, cognitive demands may be underestimated when using performance measures, highlighting the need to interpret them alongside subjective and physiological measures.

## 5. Consequences of Poor Cognition

During surgery, surgeons continuously integrate procedural, visual, and auditory stimuli and information, while balancing physical tasks. When the cumulative demands of these tasks exceed cognitive capacity, the surgeon may experience cognitive overload, reducing judgement, impairing technical skills, and deteriorating performance [37].

Cognitive workload is influenced by different interacting levels, including the task itself, the operator, and the environment [36]. Both high and low cognitive workload states are associated with errors, with the latter relating to inattention or environmental distractions [36]. Up to one-third of surgical errors can be linked to excessive workload and fatigue [64]. A systematic review further identified greater stress levels being associated with poorer technical performance, alongside impaired non-technical skills such as communication and decision-making [65].

Fatigue accumulates over extended periods of continuous work, negatively impacting fine motor control, precision, and overall performance [66]. After approximately four hours of surgery, fatigue can present as mental exhaustion, irritability, and impaired clinical judgement [67,68,69]. A study found that after two hours, there was a significant decline in surgical performance, with continued subsequent deterioration [32]. Furthermore, studies have demonstrated that increased workload is associated with reduced surgical performance and increased potential for errors, with additional findings suggesting that longer operative duration is linearly associated with increased workload scores [70,71].

## 6. Strategies to Improve Cognitive Ergonomics

### 6.1. Surgeon-Level Strategies

The current literature suggests the potential of intraoperative microbreaks to improve mental focus and cognitive fatigue. A study implemented targeted stretch microbreaks (TSMB) intraoperatively and found that a 1.5 min TSMB reduced pain and improved mental focus without extending operative time [72]. The suggested implementation of TSMB supports the surgeon’s wellbeing and is an ergonomic strategy for performance and safety intervention [72]. However, the mental focus improvement was measured subjectively rather than physiologically, making results prone to recall bias [72]. Similarly, a study found that 88% of surgeons self-reported improvement or no change in mental focus by implementing microbreaks with exercises [73]. In addition, 87% of surgeons wanted to incorporate microbreaks with exercises, showcasing high surgeon acceptance [73]. A total of 73% of this cohort reported no distractions caused by these breaks [73]. Additionally, a systematic review of 32 papers found that 5 min microbreaks every 30 min improved cortisol, concentration, attention, musculoskeletal strain, physicians’ responses to stressful intraoperative events, fatigue, and stress [74]. For cognition specifically, approximately 1 min to 2 min breaks were required to influence mental performance [74]. However, this review also highlights that currently, there is no standardised definition of a “rest break” and its duration, making it difficult to confidently conclude whether rest breaks truly improve cognitive workload [74]. Table 2 shows the summary of strategies to improve cognitive ergonomics.

Despite promising advantages, there are barriers to the implementation of intraoperative breaks. A study found that surgeons easily forget to take breaks, highlighting the importance of having a reliable reminder mechanism as prompts to rest [75]. Further studies also found that intraoperative breaks did not improve heart rate, and fatigue was related to the duration of the procedure, without being affected by the break implementation [76].

### 6.2. Interface and System Design

Alongside the complexity and duration of operative tasks, the design of the console interface and system influences the surgeon’s cognitive workload. The more recent transition from traditional two-dimensional display to the 3D binocular display of the da Vinci^®^ Surgical System has restored natural depth perception, reducing the cognitive effort required for tissue discrimination, and improving surgical comfort and confidence [77]. However, display latency remains a complex liability, as delays exceeding approximately 400 milliseconds between instrument movement and visual feedback deteriorates surgical performance, and increases error rates, fatigue, and operative time [78].

The absence of haptic feedback in early RAS platforms increases cognitive demands [79]. The addition of haptic feedback was found to be associated with shorter operative time and significantly lower workload in simulated procedures [80]. Participants also reported lower physical demand, required effort, and overall improved self-perceived performance [80]. In addition, design constraints in RAS platforms may disadvantage surgeons with certain physique [81]. Data suggest that physicians outside of the height range 162 centimetres to 185 centimetres may need to adopt non-neutral postures to visualise the display and use the pedal controls, increasing the risk of musculoskeletal injuries [81]. Shorter surgeons will need to elevate seat height, compromising foot pedal access [81]. Taller surgeons may need to flex their trunk and neck to maintain eye–robot binocular connection [81]. Furthermore, current closed robotic consoles require surgeons to maintain a fixed posture which can contribute to fatigue and limit communication with the operating team [82].

Increasing the degrees of freedom of robotic instruments can mimic the natural movements of the human hand, reducing task completion time by approximately 40%, improving suturing, simplifying complex movements, and allowing attention to higher-level decision-making [83]. Motion scaling also reduces large hand movements into smaller instrument movements, which can improve precision by approximately 20–30%, therefore reducing the mental demands associated with controlling delicate movements [84]. Similarly, the development of wearable motion sensors, haptic devices, and smart garments can replicate natural hand movements rather than relying on the current rigid mechanical controllers, reducing both cognitive and physical demands in RAS [85]. Additionally, newer systems with intuitive camera interfaces, such as eye-tracking and simultaneous camera and instrument control, can reduce workflow disruptions and associated cognitive workload [26].

### 6.3. Operating Room Workflow Optimisation

During RAS, the surgeon sits at a remote console, leading to physical separation from the patient and the rest of the surgical team [86,87]. The surgeon’s view is limited to a closed console, while the team members’ views are obscured by the robotic instruments [86,87]. Therefore, the team must adapt and speak more explicitly rather than relying on non-verbal cues, increasing demands due to disruption of the standard interpersonal structure [88]. Surgeons operating from the console must compensate using clearer verbal communication, increasing both intrinsic and extraneous cognitive load [89].

Instrument changes, the docking process, and anticipation to the changing needs of the surgeon also introduces increased mental workload for the operative team [90]. Furthermore, once the robot is docked, patient repositioning becomes challenging [91]. Integrated operating tables counteract this by allowing patient repositioning without undocking the robot, maintaining a continuous workflow [92]. Team training programmes that are focused on communication, coordination, pre-briefings and debriefings, checklists, workflow redesign, and operating workspace modifications have potential to improve safety and efficiency [88]. Short training sessions and real-time coaching are practical solutions given the limited time within hospital settings [88]. However, such checklists may be ignored or cause frustration when poorly designed, emphasising the importance of using a collaborative approach with hospital staff when designing strategies [88].

### 6.4. Artificial Intelligence and Decision Support

Currently, robotic systems such as the da Vinci^®^ Surgical System are exclusively controlled by humans [10]. The robot translates the surgeon’s movements into instrument manoeuvres; therefore, patient outcomes are directly affected by surgeon factors such as skill, experience, and decision-making [10].

AI implementation can reduce task duration and physical workload, thereby improving ergonomics [10]. AI models can also analyse the surgeon’s workload by analysing physiological signals with 65% to 79% accuracy [48]. Currently, AI provides assistance and automation, improving the surgical view by removing blur, improving colour, and reducing visual noise [10,93]. AI-assisted haptic feedback systems can measure the force applied to tissues, warn surgeons regarding force, and provide vibration or visual feedback.

Surgical decision-making is impacted by time constraints, uncertainty, cognitive bias, and decision fatigue [94]. Future AI developments can also improve navigation, analysis, and visual guidance, reducing the cognitive effort required to interpret the operative field [85]. For example, repetitive tasks such as suturing or tissue manipulation may become partially automated by integrating AI into robotic systems [95]. Additionally, AI in RAS can reduce the cognitive burden associated with processing large quantities of patient information by analysing electronic health records to provide real-time risk predictions and treatment recommendations [94]. The integration of virtual fixtures creates boundaries to guide the surgeon’s instruments along safe paths, preventing injuries to nearby anatomical structures, and reducing working memory and cognitive workload [96]. However, the overall implementation of AI has limitations that can paradoxically increase cognitive workload. For instance, alarm fatigue may occur when the team is exposed to excessive AI-generated alerts, increasing extraneous load [97]. Additionally, AI usage requires careful ethical considerations, such as inequality, overdependence, confidentiality concerns, and the mimicking of human biases [98].

### 6.5. Training and Education

Cognitive training can improve attention, working memory, problem-solving skills, and self-confidence [99]. With repetition, information in the brain becomes organised into schemas, which are automated mental frameworks [2]. This automation allows cognition to be directed towards complex aspects of a task and reduces demands on working memory [2]. With further practice, these schemas start integrating multiple elements simultaneously, reducing intrinsic cognitive workload [2]. Traditional cognitive training methods in surgery include mentorship, technical practice, and video-based learning [100]. In other high-performance occupations, such as aviation and sports, mental rehearsal, cognitive task analysis, and computer-based simulations are used to help improve attention, memory, problem-solving skills, and confidence [99]. A study found that participants allocated to a simulator training group on the da Vinci^®^ Surgical System had a 16% improvement across training modules [99]. The cognitive training group improved by 25%; however, it was noted that this improvement in skill was not transferrable to the robotic simulator [99].

In RAS, the dual-console system allows real-time supervision, assessment, and feedback, as an experienced surgeon observes and provides feedback to the trainee in a supervised environment [101]. Video-based learning supports skill refinement, efficiency, and reflective efforts, with evidence suggesting that video-based teaching is superior to textbook learning [102]. One study showed that while simulation-based training improves robotic surgical skills, mentoring reduces cognitive workload and improves performance [103].

Mental rehearsal and training involves deliberately practising the same motor tasks without physical execution [104]. This activates neural pathways that drive neuroplastic changes in the brain, allowing for basic skill acquisition, with subsequent motor skills improved by physical practice [104]. A systematic review further highlights that given the surgical training constraints, such as reduced working hours, increased patient outcome audits, and complexity of novel minimally invasive procedures, training models must expand beyond traditional simulation-based systems to optimise evaluation [104].

Cognitive task analysis (CTA) is an approach to cognitive training which captures how surgeons think during a procedure by either interviewing them or observing them to understand their decision-making and problem-solving skills, as well as their stepwise thinking [105]. CTA has been found to improve technical skills, decision-making, task complication, and skill retention [105]. A meta-analysis found that CTA-based training provides surgeons with a structured understanding of their own cognitive processes and procedural steps, improving technical performance more effectively than traditional training methods [106].

Simulation-based cognitive training (SBCT) combines cognitive training with simulators [105]. An example is the use of virtual reality or computer-based programmes, which allows surgeons to practice movements in parallel with thinking skills [105]. While traditional simulation training techniques focus on improving motor skills, SBCT targets executive functions that are required in high-performance occupations such as surgery [105]. Simulation-based cognitive training through an app has also been developed which can be used in conjunction with the da Vinci^®^ Surgical System [107].

## 7. Robotic Colorectal Surgery: An Example of the Interplay Between Technology and Cognitive Ergonomics

In colorectal surgery particularly, there are unique sets of cognitive ergonomic considerations. In robotic colorectal surgery, visualisation is superior compared to conventional laparoscopic surgery. This is brought about by the 3D vision, stable control of the camera and retracting robotic arm and a constant line-of-sight view [28]. The robotic surgeon can also invert the view of the camera 180 degrees upwards to improve visualisation during posterior dissection of the rectum as it turns the corner towards the pelvic floor. These are all important aspects of enabling good views especially deep in the pelvis during rectal dissection.

On the other hand, there can be detrimental visualisation ergonomics associated with RAS. Studies to date suggest that robotic colorectal surgery is associated with longer operative time compared to laparoscopic techniques, particularly in the early part of the learning curve [108]. This can lead to visual fatigue associated with prolonged constant viewing of the video display. This results in computer vision syndrome which includes physical symptoms such as dry eyes, eyestrain and headache [109]. Visual cues can be used to compensate for absence of haptic feedback. In colorectal surgery, there is a regular need to retract bowel and mesentery in different directions. Surgeons operating on the robotic platform have to constantly employ visual cues such as tissue deformation when retracting to avoid excessive force being applied [110]. Over the course of a long complex case, the constant need to engage visual cues to avoid damaging tissue can add to cognitive fatigue.

While robotic surgery excels in a targeted operative field, seen in gynaecological and urological surgeries, it possesses unique challenges in multi-quadrant surgery seen in colorectal procedures. This can range from two-quadrant access (low anterior resection with splenic flexure mobilisation) to four-quadrant access (subtotal or total proctocolectomy) [111]. To enable access to all four quadrants utilising a closed-console robotic system such as the da Vinci^®^ Xi model, re-docking as well as boom rotation is required [111,112].

Operating away from the targeted anatomy, as seen in multi-quadrant surgery, results in robot arm clashes which restrict movement and impair control of instruments [110]. To mitigate clashing, surgeons can move the position of the camera, hence changing the number of left- versus right-hand instruments. This results in disruption to the flow of surgery as well as increasing the surgeon’s cognitive load.

Laparoscopic surgery, especially in restricted space such as the pelvis, often results in poor ergonomic arm positioning of the surgeons and assistants. Surgeons are often required to maintain unnatural and uncomfortable postures for prolonged periods resulting in muscle fatigue [113]. As muscle fatigues, the surgeon must exert more effort in order to maintain a given muscle contraction [113]. Factors that contribute to this include limited freedom of movement, limitations in instrument design and poor positioning of the monitors [114]. In rectal surgery, the surgeon often must maintain an uncomfortable position for extended periods (for retraction and dissection), especially during total mesorectal dissection or when performing lateral node dissection. This, together with the already complex anatomy of the pelvis, quickly adds cognitive demand in laparoscopic colorectal surgery. The seated robotic surgeon console, endo-wristed instruments and clutch mechanism overcome some of these limitations [113].

While Indocyanine Green (ICG) is available in certain laparoscopic platforms, it is fully integrated into the da Vinci^®^ robotic platform as the Firefly mode. While widely used to assess perfusion in colorectal surgery, it can also be used to identify structures such as the ureter, for lymphatic mapping and as a tattoo for intraoperative identification of the lesion [115]. Ureteric injury is a rare but morbid complication of colorectal surgery. Locating the left ureter can be stressful in locally advanced rectal cancer, complicated diverticulitis, and in a previously irradiated pelvis [115]. Failure to identify the ureter adds to the surgeon’s cognitive workload considerably. Studies showed that intraurethral ICG is a safe and effective method of intraoperative uretheric identification [116]. The use of India Ink to mark colorectal lesions in minimally invasive surgery is well established. In rectal cancer, knowing the exact location of the tumour is crucial in securing adequate distal margin, which has implications on local recurrence. India Ink, when injected into the rectum, disrupts the TME dissection plane and makes surgery difficult [115,117]. The use of endoscopic clips with ICG resin allows precise localisation of tumour location, hence removing the uncertainty of distal margin during robotic rectal surgery [117].

## 8. Future Directions

Technological developments have the potential to reduce intraoperative cognitive burden, when human factors are factored into design considerations. Transition to real-time monitoring of the surgeon’s workload, alongside associated recommendations and adjustments, can transform AI into a co-pilot [10]. Additionally, augmented reality (AR) and virtual reality (VR) technologies can support surgical planning, simulation, education, and skills training, improving intraoperative performance [93]. Further prospective studies must explore the cognitive implications of AR and VR incorporation into surgical units. Telesurgery can potentially improve healthcare access in regional areas, support regional training, and allow surgical performance without requiring presence in the same operating room [118]. However, signal latency remains a significant technical barrier to safe long-distance surgeries at the current time [119]. A study highlighted the potential role of neurocognitive monitoring to measure cognitive workload during telesurgery [11]. Changes in intraparietal sulcus activity were able to identify the effects of communication delays, even when the surgeons were not consciously aware of deteriorating surgical performance, suggesting the use of neurocognitive monitoring to understand cognitive demands during RAS [11].

## 9. Research Gaps

### 9.1. Lack of Standardised Measurement Tools for Cognitive Load

Most studies rely on subjective measurement tools with limited available real-time data. Research that combines physiological tracking with subjective tools can capture cognitive workload fluctuations and compare personal surgical experiences with physiological markers. Future research needs to be oriented towards empirical studies that evaluate how the combination of physical and cognitive workload impacts patient outcomes. Longitudinal studies with the integration of physiological measures to improve accuracy and sensitivity should be conducted in real operative environments to understand the dynamic behaviour and fluctuations in both physical and cognitive workloads. The outlined studies emphasise that when subjective tools are used, cognitive workload differences between laparoscopic and RAS procedures often fail to reach significance; however, when physiological markers are used, there is a difference in cognitive effort [46,120,121].

### 9.2. Impact on Patients

There is a lack of data relating the impact of the surgeon’s cognitive workload and both short-term and long-term patient outcomes. Current data does not reflect the cognitive fatigue that may accumulate chronically in RAS surgeons. Additionally, the correlation between surgical workload and patient outcomes, complication rates, and impacted long-term outcomes, is essentially undocumented. Prospective cohort studies that correlate surgical workload with patient outcomes can provide a clinical framework and emphasise impacts on both surgeons and patients to guide ergonomic designs.

### 9.3. Interaction Between Physical and Cognitive Ergonomics

Current evidence regarding the impact of RAS on cognitive workload remains inconsistent. There is limited research exploring the bidirectional relationship between physical and cognitive workload. Both cognitive and physical workload is individually characterised; however, the interaction between the two remain simulation-based or methodologically heterogeneous, with limited longitudinal evidence [122]. For ergonomic design and interventions, future research must measure physical and cognitive workload together rather than individual characters, using a multimodal monitoring system in real-time procedures [6].

## 10. Conclusions

While RAS is a major advancement in modern surgery, the shift from physical to cognitive demands has its own implications for surgical performance and patient safety. Although many studies focus on subjective measures, the introduction of physiological measurement methods now provides real-time monitoring. Future studies should focus on how this increase in cognitive load affects patient outcomes, as well as interventions to mitigate this.

## Figures and Tables

**Table 1:** Summary of differences between cognitive workload measurement techniques in surgery.

Measurement Tool	Category	Advantages	Limitations
NASA Task Load Index (NASA-TLX)	Subjective, self-report	Easy to implement, widely used, extensive research available	Retrospective, subjective to recall bias
Surgery Task Load Index (SURG-TLX)	Subjective, self-report	Easy to implement, surgery-specific	Retrospective, subjective to recall bias
Electroencephalogram (EEG)	Neurophysiological	100% superiority in detecting cognitive demand, can differentiate between different sources of load	Requires extensive electrode set-up, physical interference with console use, uncomfortable, distracting
Functional Near-Infrared Spectroscopy	Neurophysiological	Portable, safe, wearable	Low temporal resolution, confounded by physiological changes, susceptible to motion artefacts
Eye Tracking	Physiological, Behavioural	Real-time data, non-invasive	Interpretation is complex, validity concerns, influenced by external environmental factors
Salivary cortisol, Salivary amylase	Biochemical biomarkers	Neuroendocrine data provided, objective	Influenced by external environmental factors
Surgical Performance Metrics	Objective	Objective, standardised outcome measures, patient outcomes measured	Fail to capture subjective and subconscious experiences of the surgeon
Heart Rate Variability (HRV)	Physiological	Continuous, non-invasive, real-time data	Insensitive to primary task differences, confounded by internal and external factors

**Table 2:** Summary of strategies to improve cognitive ergonomics.

Category	Strategy	Mechanism for Reducing Cognitive Workload	Advantages	Limitations
Interface and system design improvements	3D visualisation	Restores depth perception	Improved surgeon comfort and visual interpretation of the surgical field.	Display latency can increase errors and operative time
	Haptic feedback restoration	Restores tactile sensation	Reduces self-perceived workload, physical demands, and operative time.	Availability depends on technological development
	Console design	Improve surgeon’s posture	Improved ergonomic design and wearable interfaces may reduce fatigue.	Surgeons outside average height ranges are disadvantaged when using existing consoles
	Instrument and camera optimisation	Provide motion scaling and increased instrument degrees of freedom	Improve efficiency and accuracy. Allow surgeon to focus on complex decision-making.	Availability depends on technological development
Operating room workflow optimisation	Team communication and workflow redesign	Improved communication and teamwork	Reduces extraneous workload and improves workflow efficiency.	Limited evidence regarding effects on cognitive workload
Surgeon-level strategies	Intraoperative microbreaks	Recover cognitive resources and reduce cognitive fatigue	Decreasing stress, improving concentration and focus.	Current evidence is based primarily on subjective measures
	Cognitive training	Reduces intrinsic cognitive load and working memory by developing automaticity	Improves focus, working memory, technical functioning, and confidence.	Only in simulated studies
	Mentorship and training	Provides immediate feedback during operations	Improves technical performance and learning efficiency.	Limited evidence
	Cognitive task analysis (CTA) and mental rehearsal	Improves planning and decision-making	Improves technical performance, decision-making, and long-term skill retention.	Application in robotic surgery is limited
Artificial intelligence (AI)	AI-assisted visual enhancement, haptic feedback, and decision-making systems	Improves image quality, provides force estimation, and provides risk predictions, treatment recommendations, and procedural guidance	Reduces surgeon’s cognitive workload.	Requires further validation in real-life setting, confidentiality concerns, risk of overreliance

## Data Availability

Data is available on request to authors.
